# (*S*)-Benzyl 3-(4-hy­droxy­phen­yl)-2-(trityl­amino)­propano­ate

**DOI:** 10.1107/S1600536811017351

**Published:** 2011-05-14

**Authors:** Meimei Chen, Xinmei Lai, Changen Zhou, Xuemei Yang

**Affiliations:** a College of Traditional Chinese Medicine, Fujian University of Traditional Chinese Medicine, Fuzhou, 350108 Fujian, People’s Republic of China

## Abstract

The title compound, C_35_H_31_NO_3_, was obtained by the reaction of (*S*)-benzyl 2-amino-3-(4-hy­droxy­phen­yl)propano­ate and (chloro­methane­tri­yl)tribenzene. The enanti­omer has been assigned by reference to an unchanging chiral centre in the synthetic procedure. In the crystal, mol­ecules are linked into chains running along the *a* axis by inter­molecular O—H⋯O hydrogen bonds.

## Related literature

For the synthesis and the physiological role of isodityrosine, see: Skaff *et al.* (2005 ▶). For the structure of the NH_2_ analogue of the title compound, (*S*)-benzyl 2-amino-3-(4-hydroxyphenyl)propanoate, see: Luo *et al.* (2009 ▶).
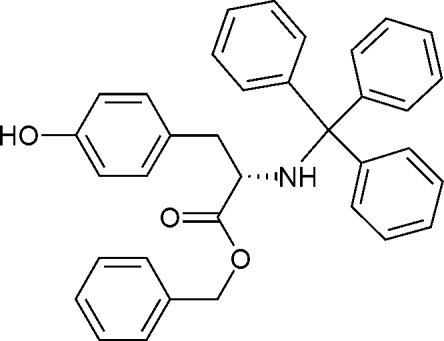


## Experimental

### 

#### Crystal data

C_35_H_31_NO_3_
*M* *_r_* = 513.61
Orthorhombic, 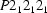

*a* = 9.1188 (18) Å
*b* = 15.774 (3) Å
*c* = 19.393 (4) Å
*V* = 2789.4 (10) Å^3^
*Z* = 4
Mo *K*α radiation
μ = 0.08 mm^−1^
*T* = 293 K
0.32 × 0.25 × 0.11 mm

#### Data collection

Rigaku Mercury CCD diffractometer
Absorption correction: multi-scan (*ABSCOR*; Higashi, 1995 ▶) *T*_min_ = 0.976, *T*_max_ = 0.991
24111 measured reflections
3097 independent reflections
2200 reflections with *I* > 2σ(*I*)
*R*_int_ = 0.053

#### Refinement

*R*[*F* ^2^ > 2σ(*F* ^2^)] = 0.041
*wR*(*F* ^2^) = 0.093
*S* = 1.09
3097 reflections
356 parameters
H atoms treated by a mixture of independent and constrained refinement
Δρ_max_ = 0.10 e Å^−3^
Δρ_min_ = −0.14 e Å^−3^

### 

Data collection: *CrystalClear* (Rigaku, 2000 ▶); cell refinement: *CrystalClear*; data reduction: *CrystalClear*; program(s) used to solve structure: *SHELXS97* (Sheldrick, 2008 ▶); program(s) used to refine structure: *SHELXL97* (Sheldrick, 2008 ▶); molecular graphics: *SHELXTL* (Sheldrick, 2008 ▶); software used to prepare material for publication: *SHELXTL*.

## Supplementary Material

Crystal structure: contains datablocks I, global. DOI: 10.1107/S1600536811017351/zq2100sup1.cif

Structure factors: contains datablocks I. DOI: 10.1107/S1600536811017351/zq2100Isup2.hkl

Supplementary material file. DOI: 10.1107/S1600536811017351/zq2100Isup3.cml

Additional supplementary materials:  crystallographic information; 3D view; checkCIF report

## Figures and Tables

**Table 1:** Hydrogen-bond geometry (Å, °)

*D*—H⋯*A*	*D*—H	H⋯*A*	*D*⋯*A*	*D*—H⋯*A*
O3—H3*A*⋯O1^i^	0.82	1.95	2.772 (3)	175

Symmetry code: (i) 
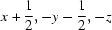
.

**Fig. 1.**
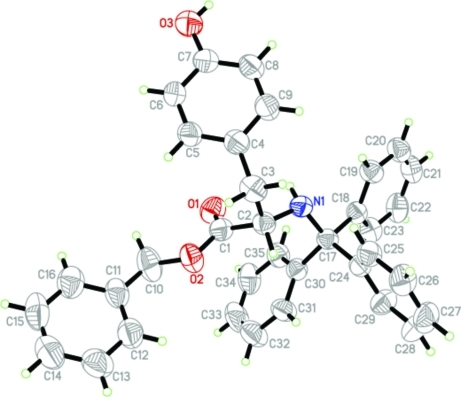
The molecular structure of the compound with 50% probability displacement ellipsoids (arbitrary spheres for H atoms).

## supplementary crystallographic information

### Comment

The title compound is an important intermediate in the synthesis of isodityrosine, which occurs in plant cell wall proteins and presumably conveys a strengthening and/or defensive role to the proteins (Skaff *et al.*, 2005). The molecular structure of the title compound is shown in Fig. 1. The bond lengths and angles in the compound are comparable to those reported for a similar compound (Luo *et al.*, 2009). The dihedral angle between the C18-phenyl and C24-phenyl, C18-phenyl and C30-phenyl, C24-phenyl and C30-phenyl planes are 80.2 (1), 61.9 (1) and 65.4 (1)°, respectively. The crystal packing is stabilized by strong O—H···O intermolecular hydrogen-bonding interactions involving the hydroxyl group which link the molecules into a chain running along the *a* axis (Table 1).

### Experimental

To a solution of (*S*)-benzyl 2-amino-3-(4-hydroxyphenyl)propanoate (0.68 g, 2.5 mmol), and (chloromethanetriyl)tribenzene (0.70 g, 2.5 mmol) in acetonitrile (8 ml) at 273 K was added dropwise triethylamine (0.40 g, 4 mmol). The cooling bath was removed and the mixture warmed to ambient temperature for 2 h. The solvent was removed and the crude product was purified by column chromatography (petroleum ether-ethyl acetate, 4:1) to give the title compound (I) as a white solid in 85% yield. Single crystals of (I) were obtained by slow evaporation of a petroleum ether/ethyl acetate solution (6:1 *v*/*v*).

### Refinement

The NH hydrogen atom was located in a difference Fourier map and freely refined. All other H atoms were placed in geometrically idealized positions and constrained to ride on their parent atoms, with C—H = 0.93 Å (aromatic), 0.97 Å (methylene), 0.98 Å (methine), O—H = 0.82 Å, and *U*_iso_(H) = 1.2*U*_eq_(C) and 1.5*U*_eq_(O). In the absence of significant anomalous scattering effects, Friedel pairs were merged. The absolute configuration of (I) was assigned assuming that the absolute configuration of the starting materials was retained during the synthesis.

### Crystal data

**Table:**

C_35_H_31_NO_3_	*F*(000) = 1088
*M**_r_* = 513.61	*D*_x_ = 1.223 Mg m^−^^3^
Orthorhombic, *P*2_1_2_1_2_1_	Mo *K*α radiation, λ = 0.71073 Å
Hall symbol: P 2ac 2ab	Cell parameters from 1875 reflections
*a* = 9.1188 (18) Å	θ = 3.3–27.5°
*b* = 15.774 (3) Å	µ = 0.08 mm^−^^1^
*c* = 19.393 (4) Å	*T* = 293 K
*V* = 2789.4 (10) Å^3^	Plate, colourless
*Z* = 4	0.32 × 0.25 × 0.11 mm

### Data collection

**Table:**

Rigaku Mercury CCD diffractometer	3097 independent reflections
Radiation source: sealed tube	2200 reflections with *I* > 2σ(*I*)
graphite	*R*_int_ = 0.053
φ and ω scans	θ_max_ = 26.0°, θ_min_ = 3.1°
Absorption correction: multi-scan (*ABSCOR*; Higashi, 1995)	*h* = −9→11
*T*_min_ = 0.976, *T*_max_ = 0.991	*k* = −19→19
24111 measured reflections	*l* = −23→23

### Refinement

**Table:**

Refinement on *F*^2^	Primary atom site location: structure-invariant direct methods
Least-squares matrix: full	Secondary atom site location: difference Fourier map
*R*[*F*^2^ > 2σ(*F*^2^)] = 0.041	Hydrogen site location: inferred from neighbouring sites
*wR*(*F*^2^) = 0.093	H atoms treated by a mixture of independent and constrained refinement
*S* = 1.09	*w* = 1/[σ^2^(*F*_o_^2^) + (0.0476*P*)^2^] where *P* = (*F*_o_^2^ + 2*F*_c_^2^)/3
3097 reflections	(Δ/σ)_max_ < 0.001
356 parameters	Δρ_max_ = 0.10 e Å^−^^3^
0 restraints	Δρ_min_ = −0.14 e Å^−^^3^

### Special details

**Table:**

Geometry. All e.s.d.'s (except the e.s.d. in the dihedral angle between two l.s. planes) are estimated using the full covariance matrix. The cell e.s.d.'s are taken into account individually in the estimation of e.s.d.'s in distances, angles and torsion angles; correlations between e.s.d.'s in cell parameters are only used when they are defined by crystal symmetry. An approximate (isotropic) treatment of cell e.s.d.'s is used for estimating e.s.d.'s involving l.s. planes.
Refinement. Refinement of *F*^2^ against ALL reflections. The weighted *R*-factor *wR* and goodness of fit *S* are based on *F*^2^, conventional *R*-factors *R* are based on *F*, with *F* set to zero for negative *F*^2^. The threshold expression of *F*^2^ > σ(*F*^2^) is used only for calculating *R*-factors(gt) *etc*. and is not relevant to the choice of reflections for refinement. *R*-factors based on *F*^2^ are statistically about twice as large as those based on *F*, and *R*- factors based on ALL data will be even larger.

### Fractional atomic coordinates and isotropic or equivalent isotropic displacement parameters (Å^2^)

**Table:**

	*x*	*y*	*z*	*U*_iso_*/*U*_eq_	
O1	−0.8415 (2)	−0.39107 (11)	−0.11380 (10)	0.0635 (5)	
O2	−0.88195 (19)	−0.52988 (11)	−0.09751 (11)	0.0642 (5)	
O3	−0.5931 (3)	−0.20753 (13)	0.11146 (12)	0.0932 (7)	
H3A	−0.5162	−0.1806	0.1105	0.140*	
N1	−0.5558 (2)	−0.42813 (13)	−0.16568 (11)	0.0470 (5)	
H1A	−0.580 (3)	−0.3773 (16)	−0.1508 (14)	0.055 (7)*	
C1	−0.7971 (3)	−0.46331 (15)	−0.11394 (14)	0.0523 (6)	
C2	−0.6402 (3)	−0.48944 (14)	−0.12606 (13)	0.0476 (6)	
H2A	−0.6401	−0.5434	−0.1511	0.057*	
C3	−0.5679 (3)	−0.50374 (15)	−0.05462 (14)	0.0587 (7)	
H3B	−0.6194	−0.5490	−0.0310	0.070*	
H3C	−0.4674	−0.5219	−0.0614	0.070*	
C4	−0.5684 (3)	−0.42608 (16)	−0.00941 (13)	0.0553 (6)	
C5	−0.6842 (3)	−0.41039 (18)	0.03513 (14)	0.0650 (8)	
H5A	−0.7593	−0.4501	0.0384	0.078*	
C6	−0.6923 (3)	−0.33742 (18)	0.07511 (15)	0.0680 (8)	
H6A	−0.7719	−0.3283	0.1041	0.082*	
C7	−0.5796 (3)	−0.27833 (18)	0.07110 (16)	0.0650 (8)	
C8	−0.4624 (3)	−0.29330 (18)	0.02834 (16)	0.0668 (8)	
H8A	−0.3859	−0.2544	0.0263	0.080*	
C9	−0.4573 (3)	−0.36602 (17)	−0.01186 (16)	0.0628 (7)	
H9A	−0.3778	−0.3747	−0.0411	0.075*	
C10	−1.0349 (3)	−0.5109 (2)	−0.0818 (2)	0.0829 (10)	
H10A	−1.0841	−0.4903	−0.1229	0.099*	
H10B	−1.0402	−0.4671	−0.0468	0.099*	
C11	−1.1086 (3)	−0.58942 (19)	−0.05648 (16)	0.0632 (7)	
C12	−1.0872 (3)	−0.6677 (2)	−0.08728 (17)	0.0738 (8)	
H12A	−1.0209	−0.6726	−0.1235	0.089*	
C13	−1.1629 (4)	−0.7384 (2)	−0.06509 (19)	0.0823 (10)	
H13A	−1.1461	−0.7908	−0.0856	0.099*	
C14	−1.2625 (4)	−0.7310 (3)	−0.0128 (2)	0.0860 (10)	
H14A	−1.3161	−0.7781	0.0011	0.103*	
C15	−1.2837 (4)	−0.6554 (2)	0.01881 (19)	0.0864 (10)	
H15A	−1.3504	−0.6511	0.0549	0.104*	
C16	−1.2062 (3)	−0.5841 (2)	−0.00253 (17)	0.0767 (9)	
H16A	−1.2204	−0.5326	0.0198	0.092*	
C17	−0.5633 (2)	−0.43267 (14)	−0.24205 (12)	0.0435 (5)	
C18	−0.4822 (3)	−0.35431 (13)	−0.26981 (14)	0.0460 (6)	
C19	−0.3684 (3)	−0.31829 (14)	−0.23272 (15)	0.0537 (7)	
H19A	−0.3452	−0.3397	−0.1894	0.064*	
C20	−0.2885 (3)	−0.25094 (14)	−0.25893 (17)	0.0605 (8)	
H20A	−0.2128	−0.2273	−0.2331	0.073*	
C21	−0.3209 (3)	−0.21891 (16)	−0.32313 (18)	0.0653 (8)	
H21A	−0.2666	−0.1742	−0.3410	0.078*	
C22	−0.4340 (3)	−0.25330 (16)	−0.36070 (17)	0.0675 (8)	
H22A	−0.4569	−0.2313	−0.4039	0.081*	
C23	−0.5139 (3)	−0.32054 (16)	−0.33443 (15)	0.0576 (7)	
H23A	−0.5899	−0.3435	−0.3604	0.069*	
C24	−0.4747 (2)	−0.50967 (13)	−0.26782 (14)	0.0453 (6)	
C25	−0.3816 (3)	−0.55510 (14)	−0.22560 (15)	0.0538 (6)	
H25A	−0.3730	−0.5403	−0.1794	0.065*	
C26	−0.3005 (3)	−0.62277 (16)	−0.25129 (18)	0.0659 (8)	
H26A	−0.2390	−0.6528	−0.2219	0.079*	
C27	−0.3097 (3)	−0.64575 (17)	−0.31902 (19)	0.0709 (9)	
H27A	−0.2555	−0.6913	−0.3357	0.085*	
C28	−0.4008 (3)	−0.60035 (17)	−0.36234 (17)	0.0719 (9)	
H28A	−0.4074	−0.6150	−0.4087	0.086*	
C29	−0.4819 (3)	−0.53335 (16)	−0.33723 (16)	0.0612 (7)	
H29A	−0.5426	−0.5033	−0.3670	0.073*	
C30	−0.7246 (2)	−0.43698 (14)	−0.26576 (13)	0.0465 (6)	
C31	−0.7951 (3)	−0.51343 (16)	−0.27810 (15)	0.0604 (7)	
H31A	−0.7414	−0.5635	−0.2766	0.072*	
C32	−0.9434 (3)	−0.5167 (2)	−0.29257 (18)	0.0796 (9)	
H32A	−0.9875	−0.5687	−0.3014	0.095*	
C33	−1.0252 (3)	−0.4450 (2)	−0.29411 (18)	0.0837 (10)	
H33A	−1.1252	−0.4477	−0.3032	0.100*	
C34	−0.9585 (3)	−0.3682 (2)	−0.28198 (18)	0.0754 (9)	
H34A	−1.0136	−0.3186	−0.2833	0.090*	
C35	−0.8094 (3)	−0.36430 (17)	−0.26780 (15)	0.0586 (7)	
H35A	−0.7658	−0.3120	−0.2595	0.070*	

### Atomic displacement parameters (Å^2^)

**Table:**

	*U*^11^	*U*^22^	*U*^33^	*U*^12^	*U*^13^	*U*^23^
O1	0.0675 (12)	0.0552 (10)	0.0677 (14)	0.0113 (9)	0.0054 (10)	−0.0029 (9)
O2	0.0479 (10)	0.0620 (10)	0.0828 (15)	−0.0012 (9)	0.0103 (9)	0.0059 (10)
O3	0.0862 (15)	0.0956 (14)	0.0977 (18)	−0.0321 (12)	0.0211 (13)	−0.0388 (13)
N1	0.0494 (11)	0.0434 (10)	0.0481 (13)	−0.0039 (10)	0.0001 (10)	−0.0030 (10)
C1	0.0549 (14)	0.0506 (14)	0.0514 (17)	−0.0020 (12)	0.0001 (13)	−0.0030 (12)
C2	0.0488 (13)	0.0433 (11)	0.0509 (16)	−0.0012 (11)	0.0009 (12)	0.0007 (11)
C3	0.0599 (16)	0.0560 (14)	0.0602 (18)	−0.0017 (13)	−0.0048 (14)	0.0093 (13)
C4	0.0582 (15)	0.0619 (15)	0.0457 (15)	−0.0090 (14)	−0.0059 (13)	0.0086 (13)
C5	0.0701 (19)	0.0780 (18)	0.0470 (17)	−0.0272 (15)	0.0035 (14)	0.0034 (14)
C6	0.0656 (18)	0.0870 (19)	0.0514 (18)	−0.0230 (15)	0.0126 (15)	−0.0098 (15)
C7	0.0656 (18)	0.0720 (17)	0.057 (2)	−0.0152 (15)	0.0003 (16)	−0.0074 (15)
C8	0.0600 (17)	0.0761 (18)	0.064 (2)	−0.0204 (15)	0.0011 (16)	−0.0005 (15)
C9	0.0527 (15)	0.0773 (17)	0.0584 (19)	−0.0086 (15)	0.0012 (14)	0.0024 (15)
C10	0.0475 (14)	0.088 (2)	0.114 (3)	0.0076 (16)	0.0153 (18)	0.015 (2)
C11	0.0413 (14)	0.0848 (19)	0.0636 (19)	0.0012 (14)	−0.0009 (14)	0.0096 (16)
C12	0.0581 (17)	0.100 (2)	0.063 (2)	−0.0097 (17)	0.0019 (16)	0.0024 (17)
C13	0.074 (2)	0.095 (2)	0.079 (3)	−0.0201 (18)	−0.012 (2)	−0.0012 (19)
C14	0.070 (2)	0.108 (3)	0.079 (3)	−0.023 (2)	−0.006 (2)	0.020 (2)
C15	0.0677 (19)	0.121 (3)	0.071 (2)	−0.005 (2)	0.0142 (18)	0.027 (2)
C16	0.0615 (17)	0.095 (2)	0.074 (2)	0.0083 (18)	0.0020 (17)	0.0060 (18)
C17	0.0428 (12)	0.0409 (11)	0.0468 (15)	0.0003 (11)	−0.0020 (11)	−0.0019 (11)
C18	0.0412 (12)	0.0405 (12)	0.0561 (17)	0.0037 (10)	0.0022 (12)	0.0015 (12)
C19	0.0519 (15)	0.0465 (12)	0.0629 (18)	−0.0011 (12)	−0.0019 (14)	0.0002 (13)
C20	0.0518 (15)	0.0443 (13)	0.085 (2)	−0.0030 (12)	0.0055 (16)	−0.0022 (14)
C21	0.0544 (16)	0.0441 (13)	0.097 (3)	0.0032 (13)	0.0180 (17)	0.0079 (15)
C22	0.0648 (18)	0.0657 (16)	0.072 (2)	0.0100 (15)	0.0140 (17)	0.0205 (15)
C23	0.0525 (15)	0.0582 (14)	0.0620 (19)	−0.0007 (13)	0.0019 (14)	0.0059 (14)
C24	0.0403 (12)	0.0420 (12)	0.0537 (16)	−0.0010 (10)	0.0005 (12)	−0.0016 (11)
C25	0.0480 (14)	0.0526 (14)	0.0607 (17)	0.0049 (12)	−0.0015 (13)	−0.0011 (13)
C26	0.0576 (16)	0.0586 (15)	0.082 (2)	0.0164 (13)	0.0025 (16)	0.0059 (15)
C27	0.0674 (19)	0.0568 (16)	0.089 (3)	0.0153 (15)	0.0183 (18)	−0.0065 (16)
C28	0.086 (2)	0.0665 (16)	0.063 (2)	0.0143 (17)	0.0089 (18)	−0.0134 (15)
C29	0.0652 (17)	0.0611 (14)	0.0572 (19)	0.0122 (13)	−0.0035 (14)	−0.0084 (13)
C30	0.0421 (12)	0.0490 (12)	0.0483 (15)	0.0031 (12)	−0.0008 (11)	−0.0003 (12)
C31	0.0500 (14)	0.0579 (15)	0.073 (2)	−0.0059 (13)	−0.0068 (14)	−0.0077 (14)
C32	0.0519 (17)	0.087 (2)	0.100 (3)	−0.0137 (17)	−0.0099 (18)	−0.0042 (18)
C33	0.0400 (14)	0.120 (3)	0.091 (3)	−0.0061 (19)	−0.0043 (16)	0.009 (2)
C34	0.0491 (16)	0.094 (2)	0.083 (2)	0.0195 (17)	0.0048 (17)	0.0150 (18)
C35	0.0493 (14)	0.0590 (14)	0.0674 (19)	0.0051 (13)	0.0006 (14)	0.0028 (14)

### Geometric parameters (Å, °)

**Table:**

O1—C1	1.209 (3)	C16—H16A	0.9300
O2—C1	1.342 (3)	C17—C18	1.538 (3)
O2—C10	1.459 (3)	C17—C30	1.542 (3)
O3—C7	1.369 (3)	C17—C24	1.542 (3)
O3—H3A	0.8200	C18—C19	1.385 (3)
N1—C2	1.455 (3)	C18—C23	1.392 (4)
N1—C17	1.484 (3)	C19—C20	1.385 (3)
N1—H1A	0.88 (2)	C19—H19A	0.9300
C1—C2	1.508 (3)	C20—C21	1.376 (4)
C2—C3	1.551 (4)	C20—H20A	0.9300
C2—H2A	0.9800	C21—C22	1.374 (4)
C3—C4	1.506 (4)	C21—H21A	0.9300
C3—H3B	0.9700	C22—C23	1.384 (4)
C3—H3C	0.9700	C22—H22A	0.9300
C4—C5	1.387 (4)	C23—H23A	0.9300
C4—C9	1.388 (4)	C24—C25	1.380 (3)
C5—C6	1.390 (4)	C24—C29	1.398 (4)
C5—H5A	0.9300	C25—C26	1.391 (3)
C6—C7	1.389 (4)	C25—H25A	0.9300
C6—H6A	0.9300	C26—C27	1.365 (4)
C7—C8	1.373 (4)	C26—H26A	0.9300
C8—C9	1.388 (4)	C27—C28	1.381 (4)
C8—H8A	0.9300	C27—H27A	0.9300
C9—H9A	0.9300	C28—C29	1.379 (4)
C10—C11	1.492 (4)	C28—H28A	0.9300
C10—H10A	0.9700	C29—H29A	0.9300
C10—H10B	0.9700	C30—C35	1.383 (3)
C11—C16	1.376 (4)	C30—C31	1.387 (3)
C11—C12	1.385 (4)	C31—C32	1.382 (4)
C12—C13	1.381 (4)	C31—H31A	0.9300
C12—H12A	0.9300	C32—C33	1.355 (4)
C13—C14	1.366 (5)	C32—H32A	0.9300
C13—H13A	0.9300	C33—C34	1.376 (4)
C14—C15	1.355 (5)	C33—H33A	0.9300
C14—H14A	0.9300	C34—C35	1.388 (4)
C15—C16	1.391 (4)	C34—H34A	0.9300
C15—H15A	0.9300	C35—H35A	0.9300
			
C1—O2—C10	116.1 (2)	C15—C16—H16A	119.8
C7—O3—H3A	109.5	N1—C17—C18	106.75 (19)
C2—N1—C17	118.05 (19)	N1—C17—C30	110.11 (19)
C2—N1—H1A	107.5 (17)	C18—C17—C30	112.93 (19)
C17—N1—H1A	111.0 (17)	N1—C17—C24	109.69 (19)
O1—C1—O2	123.0 (2)	C18—C17—C24	105.51 (17)
O1—C1—C2	125.1 (2)	C30—C17—C24	111.62 (19)
O2—C1—C2	111.7 (2)	C19—C18—C23	117.8 (2)
N1—C2—C1	113.7 (2)	C19—C18—C17	120.6 (2)
N1—C2—C3	110.09 (19)	C23—C18—C17	121.5 (2)
C1—C2—C3	107.7 (2)	C18—C19—C20	121.2 (3)
N1—C2—H2A	108.4	C18—C19—H19A	119.4
C1—C2—H2A	108.4	C20—C19—H19A	119.4
C3—C2—H2A	108.4	C21—C20—C19	120.0 (3)
C4—C3—C2	113.6 (2)	C21—C20—H20A	120.0
C4—C3—H3B	108.8	C19—C20—H20A	120.0
C2—C3—H3B	108.8	C22—C21—C20	119.8 (3)
C4—C3—H3C	108.8	C22—C21—H21A	120.1
C2—C3—H3C	108.8	C20—C21—H21A	120.1
H3B—C3—H3C	107.7	C21—C22—C23	120.1 (3)
C5—C4—C9	117.1 (2)	C21—C22—H22A	119.9
C5—C4—C3	120.6 (2)	C23—C22—H22A	119.9
C9—C4—C3	122.2 (3)	C22—C23—C18	121.0 (3)
C4—C5—C6	122.4 (3)	C22—C23—H23A	119.5
C4—C5—H5A	118.8	C18—C23—H23A	119.5
C6—C5—H5A	118.8	C25—C24—C29	117.5 (2)
C7—C6—C5	119.0 (3)	C25—C24—C17	122.6 (2)
C7—C6—H6A	120.5	C29—C24—C17	119.8 (2)
C5—C6—H6A	120.5	C24—C25—C26	120.9 (3)
O3—C7—C8	123.7 (3)	C24—C25—H25A	119.6
O3—C7—C6	116.7 (3)	C26—C25—H25A	119.6
C8—C7—C6	119.6 (3)	C27—C26—C25	121.0 (3)
C7—C8—C9	120.5 (3)	C27—C26—H26A	119.5
C7—C8—H8A	119.7	C25—C26—H26A	119.5
C9—C8—H8A	119.7	C26—C27—C28	119.0 (3)
C8—C9—C4	121.4 (3)	C26—C27—H27A	120.5
C8—C9—H9A	119.3	C28—C27—H27A	120.5
C4—C9—H9A	119.3	C27—C28—C29	120.3 (3)
O2—C10—C11	109.2 (2)	C27—C28—H28A	119.8
O2—C10—H10A	109.8	C29—C28—H28A	119.8
C11—C10—H10A	109.8	C28—C29—C24	121.3 (3)
O2—C10—H10B	109.8	C28—C29—H29A	119.4
C11—C10—H10B	109.8	C24—C29—H29A	119.4
H10A—C10—H10B	108.3	C35—C30—C31	117.2 (2)
C16—C11—C12	118.2 (3)	C35—C30—C17	120.3 (2)
C16—C11—C10	119.4 (3)	C31—C30—C17	122.1 (2)
C12—C11—C10	122.3 (3)	C32—C31—C30	121.4 (3)
C13—C12—C11	121.0 (3)	C32—C31—H31A	119.3
C13—C12—H12A	119.5	C30—C31—H31A	119.3
C11—C12—H12A	119.5	C33—C32—C31	120.8 (3)
C14—C13—C12	119.6 (3)	C33—C32—H32A	119.6
C14—C13—H13A	120.2	C31—C32—H32A	119.6
C12—C13—H13A	120.2	C32—C33—C34	119.2 (3)
C15—C14—C13	120.4 (3)	C32—C33—H33A	120.4
C15—C14—H14A	119.8	C34—C33—H33A	120.4
C13—C14—H14A	119.8	C33—C34—C35	120.4 (3)
C14—C15—C16	120.3 (3)	C33—C34—H34A	119.8
C14—C15—H15A	119.9	C35—C34—H34A	119.8
C16—C15—H15A	119.9	C30—C35—C34	121.1 (3)
C11—C16—C15	120.4 (3)	C30—C35—H35A	119.5
C11—C16—H16A	119.8	C34—C35—H35A	119.5
			
C10—O2—C1—O1	2.4 (4)	N1—C17—C18—C23	−154.8 (2)
C10—O2—C1—C2	177.4 (3)	C30—C17—C18—C23	−33.7 (3)
C17—N1—C2—C1	−85.4 (3)	C24—C17—C18—C23	88.5 (3)
C17—N1—C2—C3	153.6 (2)	C23—C18—C19—C20	−0.1 (4)
O1—C1—C2—N1	−24.8 (4)	C17—C18—C19—C20	176.0 (2)
O2—C1—C2—N1	160.3 (2)	C18—C19—C20—C21	−0.4 (4)
O1—C1—C2—C3	97.5 (3)	C19—C20—C21—C22	0.8 (4)
O2—C1—C2—C3	−77.4 (2)	C20—C21—C22—C23	−0.8 (4)
N1—C2—C3—C4	64.6 (3)	C21—C22—C23—C18	0.3 (4)
C1—C2—C3—C4	−59.9 (3)	C19—C18—C23—C22	0.1 (4)
C2—C3—C4—C5	90.2 (3)	C17—C18—C23—C22	−175.9 (2)
C2—C3—C4—C9	−87.6 (3)	N1—C17—C24—C25	−11.5 (3)
C9—C4—C5—C6	1.1 (4)	C18—C17—C24—C25	103.2 (2)
C3—C4—C5—C6	−176.7 (3)	C30—C17—C24—C25	−133.8 (2)
C4—C5—C6—C7	−0.8 (5)	N1—C17—C24—C29	171.5 (2)
C5—C6—C7—O3	179.9 (3)	C18—C17—C24—C29	−73.8 (3)
C5—C6—C7—C8	−0.5 (5)	C30—C17—C24—C29	49.2 (3)
O3—C7—C8—C9	−179.1 (3)	C29—C24—C25—C26	−1.1 (4)
C6—C7—C8—C9	1.4 (5)	C17—C24—C25—C26	−178.2 (2)
C7—C8—C9—C4	−1.0 (5)	C24—C25—C26—C27	0.5 (4)
C5—C4—C9—C8	−0.3 (4)	C25—C26—C27—C28	0.3 (5)
C3—C4—C9—C8	177.6 (3)	C26—C27—C28—C29	−0.5 (5)
C1—O2—C10—C11	−173.2 (3)	C27—C28—C29—C24	−0.1 (4)
O2—C10—C11—C16	139.8 (3)	C25—C24—C29—C28	0.9 (4)
O2—C10—C11—C12	−43.0 (4)	C17—C24—C29—C28	178.1 (2)
C16—C11—C12—C13	0.7 (5)	N1—C17—C30—C35	78.7 (3)
C10—C11—C12—C13	−176.5 (3)	C18—C17—C30—C35	−40.5 (3)
C11—C12—C13—C14	1.3 (5)	C24—C17—C30—C35	−159.2 (2)
C12—C13—C14—C15	−2.3 (5)	N1—C17—C30—C31	−93.8 (3)
C13—C14—C15—C16	1.3 (5)	C18—C17—C30—C31	147.0 (2)
C12—C11—C16—C15	−1.8 (5)	C24—C17—C30—C31	28.3 (3)
C10—C11—C16—C15	175.6 (3)	C35—C30—C31—C32	0.7 (4)
C14—C15—C16—C11	0.8 (5)	C17—C30—C31—C32	173.4 (3)
C2—N1—C17—C18	172.46 (18)	C30—C31—C32—C33	−1.0 (5)
C2—N1—C17—C30	49.5 (3)	C31—C32—C33—C34	0.9 (5)
C2—N1—C17—C24	−73.7 (2)	C32—C33—C34—C35	−0.5 (5)
N1—C17—C18—C19	29.2 (3)	C31—C30—C35—C34	−0.3 (4)
C30—C17—C18—C19	150.4 (2)	C17—C30—C35—C34	−173.2 (3)
C24—C17—C18—C19	−87.4 (3)	C33—C34—C35—C30	0.2 (5)

### Hydrogen-bond geometry (Å, °)

**Table:**

*D*—H···*A*	*D*—H	H···*A*	*D*···*A*	*D*—H···*A*
O3—H3A···O1^i^	0.82	1.95	2.772 (3)	175

Symmetry codes: (i) *x*+1/2, −*y*−1/2, −*z*.
